# From Insulin Measurement to Partial Exocytosis Model: Advances in Single Pancreatic Beta Cell Amperometry over Four Decades

**DOI:** 10.1021/acsmeasuresciau.4c00058

**Published:** 2024-10-10

**Authors:** Amir Hatamie, Xiulan He, Andrew Ewing, Patrik Rorsman

**Affiliations:** † Department of Physiology, Sahlgrenska Academy, University of Gothenburg, Medicinaregatan 11−13, 41390 Gothenburg, Sweden; ‡ Department of Chemistry and Molecular Biology, University of Gothenburg, Kemivägen 10, 412 96, Gothenburg, Sweden; § Department of Chemistry, Institute for Advanced Studies in Basic Sciences (IASBS), Prof. Sobouti Boulevard, PO-Box 45195-1159, Zanjan, 45137-66731, Iran; ∥ College of Chemistry, Beijing Normal University, Beijing 100875, China; ⊥ Oxford Centre for Diabetes, Endocrinology and Metabolism, University of Oxford, Churchill Hospital, Oxford OX3 7LJ, U.K.

**Keywords:** Single Cell Amperometry, Diabetes, Insulin Exocytosis, Electroanalysis, Microelectrode, Vesicle, Partial Release, Beta Cell

## Abstract

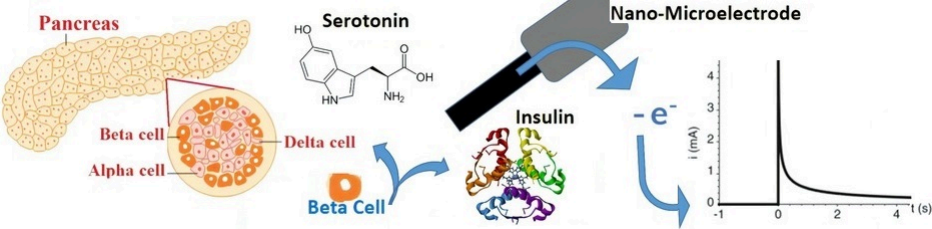

Single cell Amperometry (SCA) is a powerful, sensitive, high temporal resolution electrochemical technique used to quantify secreted molecular messengers from individual cells and vesicles. This technique has been extensively applied to study the process of exocytosis, and it has also been applied, albeit less frequently, to investigate insulin exocytosis from single pancreatic beta cells. Insufficient insulin release can lead to diabetes, a chronic lifestyle disorder that affects millions of people worldwide. This review aims to summarize and highlight electrochemical measurements of insulin via monitoring its secretion from beta cells by SCA with micro- and nanoelectrodes since the 1990s and to explain how and why serotonin is used as a proxy for monitoring insulin during exocytosis from single beta cells. Finally, we describe how the combination of SCA measurements with the intracellular vesicle impact electrochemical cytometry (IVIEC) technique has led to important findings regarding fractional release types in beta cells. These findings, reported recently, have opened a new window in the study of pore formation, exocytosis from single vesicles, and the mechanisms of insulin secretion. This sensitive cellular electroanalysis approach should help in the development of novel therapeutic strategies targeting diabetes in the future.

## Introduction

Currently, type 2 diabetes (T2D) is recognized as one of the biggest global challenges. Diabetes is estimated to affect millions of children and adults, and this number is expected to continue increasing notably. Some reports estimate it will reach 500–600 million by 2050,^1^ and this might be increased further by the COVID pandemic.^2,3^ Approximately 5–10% of diabetics have type 1 diabetes (T1D), while the remaining ∼90% have T2D. Generally, T1D results from autoimmune disturbances and genetic disorders leading to the destruction of functional β-cell mass and their function. In contrast, T2D results from defects in functional β-cell mass, abnormal insulin secretion, and reduced glucose uptake. Various factors, such as a poor lifestyle, can lead to hyperglycemia and accelerate T2D.^4,5^

Additionally, more than 100 gene variants increasing T2D risk have been identified, playing a critical role. In both types of diabetes, β-cell dysfunction in insulin production, secretion, or exocytosis is a precursor, particularly in T2D.^6,7^ Despite many investigations and findings, the exact mechanisms of T2D remain unknown, and effective drugs have not been sufficiently developed. Therefore, obtaining novel information about insulin production and secretion is very important.

**Figure 1:**
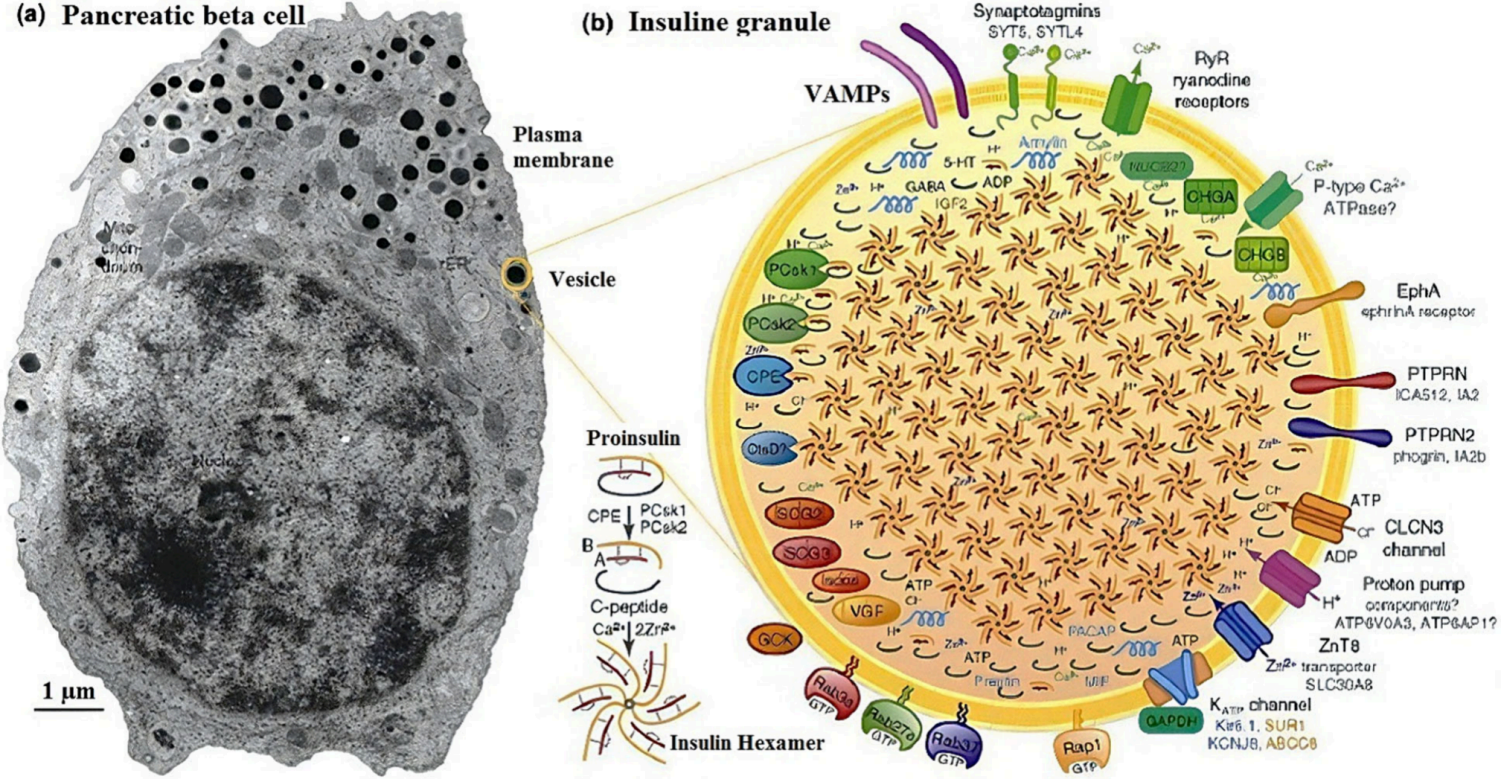
(**a**) TEM image of a single pancreatic beta cell with many vesicles (granules). (**b**) Schematic of a secretory vesicle showing the structure and components. (Reprinted from ref (^9^) copyright 2010 Elsevier).

Clearly, insulin is a key player in diabetes. Insulin plays a critical role in regulating blood glucose levels and is the only hormone responsible for controlling these levels.^4,5^ Upon synthesis of insulin as preproinsulin on the rough endoplasmic reticulum, the produced proinsulin molecules are transferred to the trans Golgi apparatus. Then, proinsulins are packaged into secretory vesicles (Approximately 200 ± 100 nm in diameter) in solid hexamer complexes with Zn ions (In the form of Ins_6_Zn_2_, an orange star-shaped structure within the vesicle (See Figure 1b)). The TEM image of a single beta cell with intracellular vesicles in the cytoplasm (Black dots) is shown in Figure 1. Normally, each β-cell contains thousands of secretory vesicles, and these nanoscale packages release their insulin cargo into the extracellular regions through the exocytosis process triggered by increasing glucose levels in the bloodstream.^8,9^ The cellular insulin secretion bioprocess is well understood and will not be described in detail here. Simply put, after the exocytosis process, these complexes (Ins_6_Zn_2_) quickly dissociate, allowing the insulin monomers to spread in the bloodstream and regulate glucose levels in our body.^5,9^ Numerous reports refer to T2D and its relation to deficiencies in the exocytosis process and the related exocytosis proteins that control and regulate insulin exocytosis from β-cells to the bloodstream.^2−7^

As seen, both the vesicles and the exocytosis process play crucial roles in insulin secretion yield, and any disturbances in either would result in impaired insulin secretion, leading to T2D. Besides these, as mentioned earlier, the SNARE (Soluble N-ethylmaleimide-sensitive factor attachment) proteins control the opening of vesicles and the exocytosis rate of insulin from single vesicles.^8−13^ This exocytosis-initiating process occurs in several steps. First, the SNARE protein groups on the cell membrane and the vesicle membrane surface interact with each other in a bioprocess called docking. After docking, during several continuous steps, these proteins can open the docked vesicles into the cell membrane and form a fusion pore. By forming the nanoscale pore, the intravesicular lumen connects to the extracellular space, allowing the vesicle content, primarily insulin, to leave the vesicle.^4,5,11^ More accurately, after pore formation and the exocytosis process, insulin is cosecreted with other components present in the secretory granules, including C-peptide, ATP, γ-aminobutyric acid (GABA), ghrelin, and amylin.^4−6^ Notably, the other components released with insulin from single vesicles play important roles in insulin secretion, insulin receptor activation, and other homeostatic effects. The exocytosis events are fast bioprocesses, occurring in milliseconds, and the pores have nanoscale diameters. Due to the fast process and small pore size, studying this subcellular process is challenging and requires sensitive optical and electrochemical analytical instruments with small dimensions and high resolution.^14−18^

During the past decade, several techniques have emerged to study and monitor insulin secretion from single β-cells from different aspects,^16,17,19−22^ allowing pre-exocytotic and postexocytotic events to be monitored at high temporal resolutions, even at the single-vesicle level. Important techniques include patch clamp,^23^ imaging,^24^ and amperometry.^19−22^ In this review, we will focus mainly on amperometric techniques with micronanoelectrodes as an excellent tool for studying the exocytosis process at beta cells from a quantitative aspect in real-time, and recent findings in this area, particularly the partial release as the preferred mode of exocytosis in beta cells.

### Single-Cell Amperometry: An Excellent Technique for Extra- and Intracellular Analysis

Currently, the main existing analytical methods for studying the exocytosis process within milliseconds include patch clamp, fluorescence electron microscopic imaging, and electrochemical techniques.^4,5,23^ In patch clamp, a small metal wire is placed in a glass micropipette filled with an electrolyte, and the glass micronanopipette is positioned into or on the cell membrane to measure the current through the membrane ion channels while the potential is clamped across the cell membrane. This allows the opening of individual ion channels to be detected by measuring conductance.^25,26^

Cell membranes behave like electrical capacitors that can be charged by applying an electrical field across the membrane. Changes in cell capacitance can indicate alterations in cell surface area. To monitor exocytosis by the patch clamp method one uses a micro or nanopipette to measure this capacitance. Monitoring capacitance enables the study of dynamic cellular phenomena, such as rapid changes in membrane surface, particularly during exocytosis and/or endocytosis.^25−28^ Specifically, since membrane capacitance is linked to surface area, an increase in capacitance is detected after vesicle docking and the formation of a fusion pore. This increase reflects an exocytosis event and the expansion of the membrane surface as the vesicle fuses with the plasma membrane, even if only transiently.^27,28^

Thus, patch clamp techniques can be used to measure not only membrane conductance but also membrane capacitance, due to the corresponding increase in surface area. As a result, even single exocytosis events can be recorded.^4,5,25−28^ Despite its high temporal resolution, which makes it valuable for measuring the dynamics of single vesicle fusion, it cannot be used to detect the identity or amount of secreted molecules during exocytosis. Fluorescence and electron microscopic imaging techniques can provide valuable information regarding the subcellular process of exocytosis, including vesicle trafficking, docking, and fusion before and after exocytosis, as well as pore formation, and can monitor secreted molecules in real time.^24,29^ Various imaging techniques, such as fluorescence microscopy, confocal fluorescence,^30,31^ total internal reflection fluorescence (TIRF),^32^ stimulated emission depletion (STED) microscopy,^33−35^ and transmission electron microscopy (TEM),^36^ have been used to study vesicles and the exocytosis process. Some of these techniques are dynamic and can monitor vesicle motion, transport, and exocytosis in single beta cells or pancreas samples in real time, while static methods like TEM (Figure 1) can be used to study vesicle size and geometry after cell and vesicle chemical fixation. Fluorescence techniques rely on fluorescent labels that can attach to vesicle or cell membrane components, whereas some use fluorescent probes that are taken up and stored inside single vesicles like false fluorescent neurotransmitters or pHluorin dyes.^37−40^ Some strategies molecularly label stored molecules within vesicles and attach to some SNARE proteins exclusively. Thus, using these labels, it is possible to monitor vesicle trafficking and pre- and postexocytosis processes in real time and conduct quantitative optical analysis after exocytosis. However, like patch clamp, quantitative analysis of secreted molecules from a single cell or vesicle is not possible during exocytosis.

Among the sensitive techniques, only electrochemistry, primarily carried out as single-cell amperometry (SCA), can analyze and determine the absolute amount of released electroactive molecules like catecholamines (dopamine, epinephrine, and norepinephrine) and other neurotransmitters like serotonin^41−50^ and reactive oxygen (ROS) and nitrogen (RNS) species.^51−53^ This is done both at and inside the cell and to measure the contents of single vesicles or during secretion from single cell into the extra cellular space. In 1990, the Wightman group began performing voltammetric studies on single adrenal chromaffin cells in vitro.^14^ They observed small and fast current peaks, which they attributed to single exocytosis release events, and detected signals due to the oxidation of released catecholamines. In fact, counting released molecules from single exocytosis events is a unique property of SCA, making it one of the most significant techniques for real-time single-cell analysis. In the following decades, this technique, along with the electrodes of different materials, sizes, and geometries used in this technique, improved and developed extensively.^46−49^

**Figure 2:**
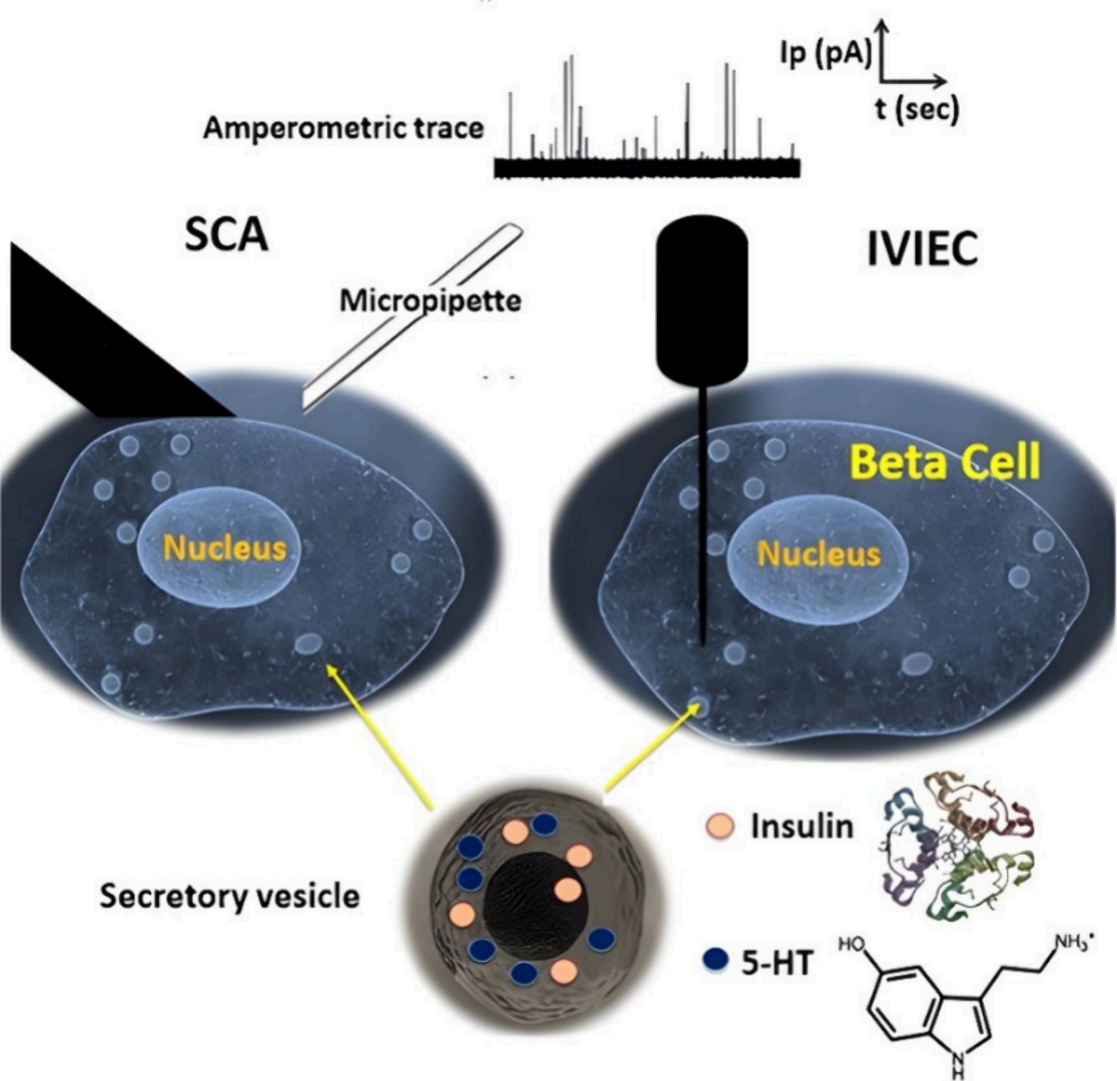
Graphical illustration of single-cell amperometry (SCA) measurements of exocytosis using a disc carbon fiber electrode (DCFE, left) positioned on the cell surface, and vesicle content analysis via IVIEC (Intracellular vesicle impact electrochemical cytometry) using a nanotip carbon fiber electrode (NCFE) placed within the cytoplasm of a single beta cell. (Reprinted from ref (^19^) copyright 2021 Wiley-VCH).

In SCA, a microelectrode, as the working electrode, mostly fabricated from bare carbon fibers, is generally placed on or very close to a single cell to perform electroanalysis at the target cell surface (Figure 2). At the same time, a single micropipette filled with a cell stimulation solution is placed next to the target cell; this pipet injects nanoliter amounts of stimulation solution around the cell, triggering exocytosis at the target cell while the working microelectrode is located on the surface (Figure 2).^44−49^ A constant potential applied between the working and reference electrodes leads to the oxidation of electroactive molecules at the working electrode. Generally, electroactive species in the vesicle are oxidized (or reduced), generating an amperometric current transient, which appears as fast spikes (Figure 2) in a plot of current versus time (I vs t). Integrating the area of these spikes gives the total charge transferred (Q), which is related to the number of molecules released (N) according to Faraday’s law.^41−45,54^ The equation is *N* = *Q*/*nF*, where N is the number of released molecules, Q is the charge from the time integral of current transients or the area of the amperometric signal, n is the number of electrons exchanged in the oxidation reaction (for example, two electrons for serotonin), and *F* is the Faraday constant (96,485 C mol^–1^ of electrons).^14,15,23,55^ Thus, based on the peak area and electron number in the reaction, the number of molecules can be estimated directly. Performing accurate quantitative analysis in real time is a unique capability of this technique. SCA has been extensively used to study exocytosis in different types of cells, yielding novel information in fields such as neuroscience and diabetes research. Recently, a new electrochemical method has been developed that is capable of directly measuring the content of vesicles before exocytosis. This technique is called intracellular vesicle impact electrochemical cytometry (IVIEC).^48,56^ Unlike SCA, IVIEC uses smaller electrodes in the nanoscale range and performs electroanalysis inside the cell to estimate vesicle content before exocytosis (Figure 2). As shown in Figure 2, the nanoscale tip electrode (such as a carbon fiber etched to a nanotip) is slowly inserted into the target cell and positioned within the cell’s cytoplasm while a suitable potential is applied. Under these conditions, intracellular vesicles in the cytoplasm come into contact with the electrode surface, causing their membranes to burst due to the applied potential—a phenomenon known as electroporation.^56^ Consequently, the vesicles expel their contents onto the electrode surface, which are then oxidized rapidly, producing amperometric signals. In IVIEC measurements, each recorded signal is quantitatively analyzed to determine the amount of stored molecules within a single vesicle.^14^

### Single Beta Cell Electroanalysis: From Direct Insulin Measurement to Serotonin Counting as an Insulin Proxy

As mentioned above, SCA is the best technique for real-time quantitative analysis during exocytosis. However, like all analytical techniques, it has some limitations. The classic version is suitable only for electroactive molecules, such as neurotransmitters including catecholamines, serotonin, histamine, and others as well as reactive oxygen species (ROS), and reactive nitrogen species (RNS).^41−53,57,58^ It cannot be used to directly analyze molecules with low or no electroactivity, like glucose and insulin. To overcome this limitation, some electrode modifications are needed, such as enzyme modification (e.g., glucose oxidase for cellular analysis of glucose) or coating the electrode with inorganic catalysts like ruthenium oxide (RuOx), which has been applied to accelerate insulin oxidation on the electrode surface.^18,23^

Generally, insulin, unlike neurotransmitters such as dopamine, exhibits lower electroactivity and cannot be detected or oxidized at bare carbon electrodes. Furthermore, the insulin molecule, which consists of 53 amino acids, is large and heavy. Notably, of these 53 amino acids, only 4 tyrosine residues can be oxidized, resulting in the production of 4 electrons.^59^ The insulin oxidation process is slow and needs to be accelerated. To address this, one effective approach is to modify the electrode surface with a metallic electrocatalyst film and particles. Another challenge is the pH of the electrochemical measurement, which is generally 7.4 (physiological pH). Most electrocatalysts show low activity at this pH and perform better in highly acidic or basic environments. In conclusion, these factors, among others, make the electroanalysis of insulin at the single-cell level and at neutral pH more challenging compared to other electroactive neurotransmitters.^60^

**Figure 3:**
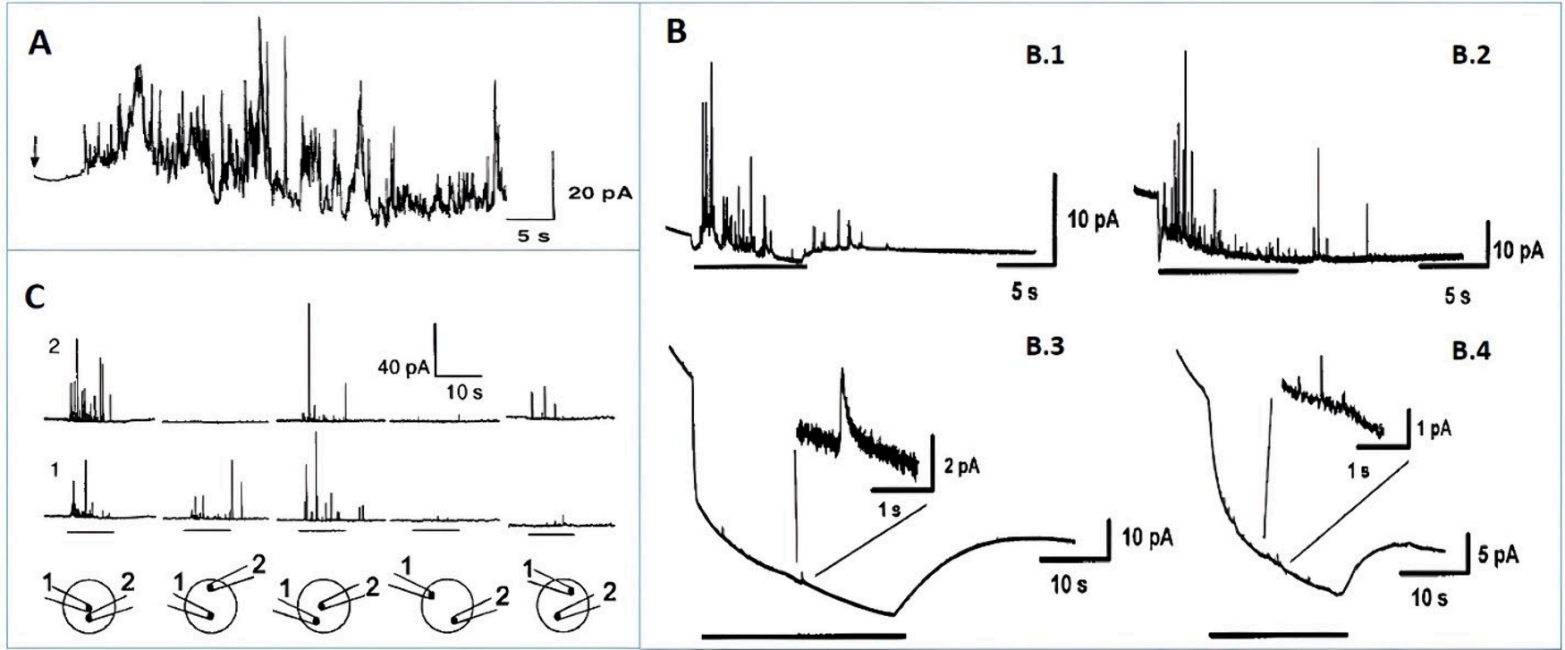
(**A**) Amperometric spike recordings from a single cell after glucose stimulation, representing the first report of insulin exocytosis measurement using a micro carbon electrode modified with a Ru-O/CN-Ru film. (Reprinted from ref (^61^) American Chemical Society copyright 1993). (**B**) Detection of insulin secretion from single beta cells of different species using a modified carbon fiber electrode with a Ru-O/CN-Ru film: (**B.1**) Canine beta cells stimulated with 200 μM tolbutamide. (**B.2**) Porcine beta cells stimulated with 200 μM tolbutamide. (**B.3**) Mouse beta cells stimulated with 30 mM K^+^. (**B.4**) INS-1 beta cells stimulated with 30 mM K^+^. (Reprinted from ref (^64^) American Chemical Society copyright 1999). (**C**) Bielectrode amperometric analysis and their recordings obtained from a single cell. The relative positions of the two electrodes (Numbered 1 and 2) are indicated by the drawings below the recordings. Note that major current spike activity was observed only when the electrodes were positioned in the middle of the cell and toward the lower left quadrant. (Reprinted from ref (^66^),\ Springer copyright 2000).

In the 1990s, Kennedy’s group fabricated and modified carbon microelectrodes with an electrocatalyst to measure secreted insulin in response to glucose stimulation from single beta cells directly (Figure 3A).^61^ They used carbon-fiber microelectrodes electrochemically modified with ruthenium compound films, such as ruthenium oxide/cyanoruthenate (Ru–O/CN-Ru) or ruthenium oxide (RuOx) separately. These compounds were selected after testing many metallic and metallic oxide catalysts. They suggested that these ruthenium compounds could act as catalysts to promote the oxidation of insulin molecules at natural physiological pH.^21,22,61−65^

After their success in measuring insulin using carbon fiber electrodes modified with ruthenium compounds in the early 1990s, they extended their research and studied the effect of extracellular Zn^2+^ levels and pH on insulin release in 1996.^22,62^ Insulin is stored inside vesicles in a complex with Zn^2+^ ions (Hexamer complex Ins_6_Zn_2_), and after exocytosis, the complex can dissociate into free insulin monomers and zinc ions in the extracellular space. The stability and dissociation of these complexes depend on extracellular pH and Zn^2+^ ion levels. They observed that decreasing the extracellular medium pH from 7.4 to around 6.5, a value close to the intravesicular pH, and increasing the level of free Zn^2+^ ions in the extracellular space could decrease the intensity of amperometric spikes in insulin measurements. Thus, they concluded that only free insulin monomers can be oxidized at the electrode surface. They suggested that under these conditions, the rate of complex dissociation due to acidic pH or high levels of free Zn^2+^ could drop, and insulin released from the Zn-insulin complex was too dilute to be detected by the electrode.

In 1997, Kennedy’s group sought to find another electrocatalyst for insulin electroanalysis,^63^ as their first reports showed low stability and poor activity of catalysts after a few experiments. They modified microelectrodes with various metallic and metallic oxide films from different metallic ions such as Ir, Ru, Rh, and Mo, and even deposited bimetallic catalysts on the electrode surface. Ultimately, they argued that electrodes with ruthenium-oxide-type catalytic films (RuOx) showed better responses than others.

In 1999, they performed insulin measurements in different beta cells from different animals and observed variations in the number and intensity of exocytosis signals.^64^ For example, in analysis of INS-1 beta cells, they observed very low and small insulin amperometric signals (Figure 3B). Apparently, inconsistent with their previous results, they suggested this to high levels of zinc in INS-1 beta cell samples.

Several groups began performing beta cell electroanalysis in the 1990s and later and found mixed results.^20,21,61−64^ Interestingly, for around two decades, they worked extensively in this area and used modified carbon fiber electrodes with ruthenium oxides as electrocatalysts. However, they later reported that these catalysts showed low stability.^20,21,61−63^ They then began testing alternative solutions. One solution tested was to analyze serotonin (5-HT) with a bare carbon fiber electrode instead of insulin to study insulin exocytosis indirectly.^64,66^

Serotonin is costored in beta cell vesicles and coreleased with insulin and is easily oxidized, unlike insulin, which cannot be detected by a bare carbon electrode. However, because pancreatic beta cell vesicles typically contain low levels of 5-HT, detecting this amount using amperometry is nearly impossible. Hence, loading vesicles by incubation with high levels of 5-HT (such as 1 mM 5-HT and 5-hydroxytryptophan, a 5-HT precursor) for a few hours before electroanalysis can elevate its concentration to easily detectable levels. This can then be used as a proxy of insulin release to study the exocytotic process in beta cells.^4,5,61−63^

As using 5-HT as an insulin proxy has become more accepted and used by researchers the use of modified electrodes for insulin measurement has not been reported again for cellular exocytosis measurements. One example, from 2000, used a 1-μm radius electrodes placed in different areas of single cells to study exocytosis distribution sites at the single-cell level.^66^ In this study, they incubated isolated beta cells with 0.5 mM 5-HT and 1 mM 5-hydroxytryptophan for 16 h and performed analysis with a bare electrode. Interestingly, as shown in Figure 3C, by placing two microelectrodes simultaneously on a single cell and chemically stimulating the cell, they found that exocytosis activities were higher in some regions and that some areas of the cells had more vesicles.

In another report in 2007, Berggren and co-workers studied 5-HT secretion from single beta cells.^20^ Based on their observations, they claimed that single pancreatic beta cells exhibited oscillations in exocytosis with a period of 1–1.5 min as measured amperometrically. They connected the timing of exocytosis with serotonin release. More recently, the Ewing and Rorsman groups^19^ treated cells with 5-HT and combined SCA with IVIEC to compare the 5-HT in vesicles with that released during exocytosis. More details on this will be presented in the next section.

### Combination of SCA and IVIEC Reveals Partial Release as the Primary Mode of the Exocytosis Process in Beta Cells

In the past decade, the development of the IVIEC technique,^14,56,57^ which allows the measurement of vesicle content before exocytosis, has opened new opportunities and led to significant findings in exocytosis monitoring. IVIEC results have introduced the concept of partial release as a common mode of exocytosis in many cell lines,^67,68^ rather than the previously accepted full release, in many neural cells and later in beta cells.

For decades, it was believed that full release was the primary mechanism of exocytosis, where each vesicle, such as an insulin vesicle, would release its entire content during a single event, with the vesicle membrane fully merging with the cell membrane. However, in the partial release process, vesicles release only a portion of their content during each exocytosis. By combining SCA with IVIEC, researchers were able to compare vesicle release to vesicle content and accurately test the hypothesis that exocytosis of single vesicles is an all-or-none process. The results revealed that most vesicles prefer to release only a portion of their content for normally considered full release in addition to kiss and run release. This challenged the long-held belief in the full release mechanism.^38,67−70^

**Figure 4:**
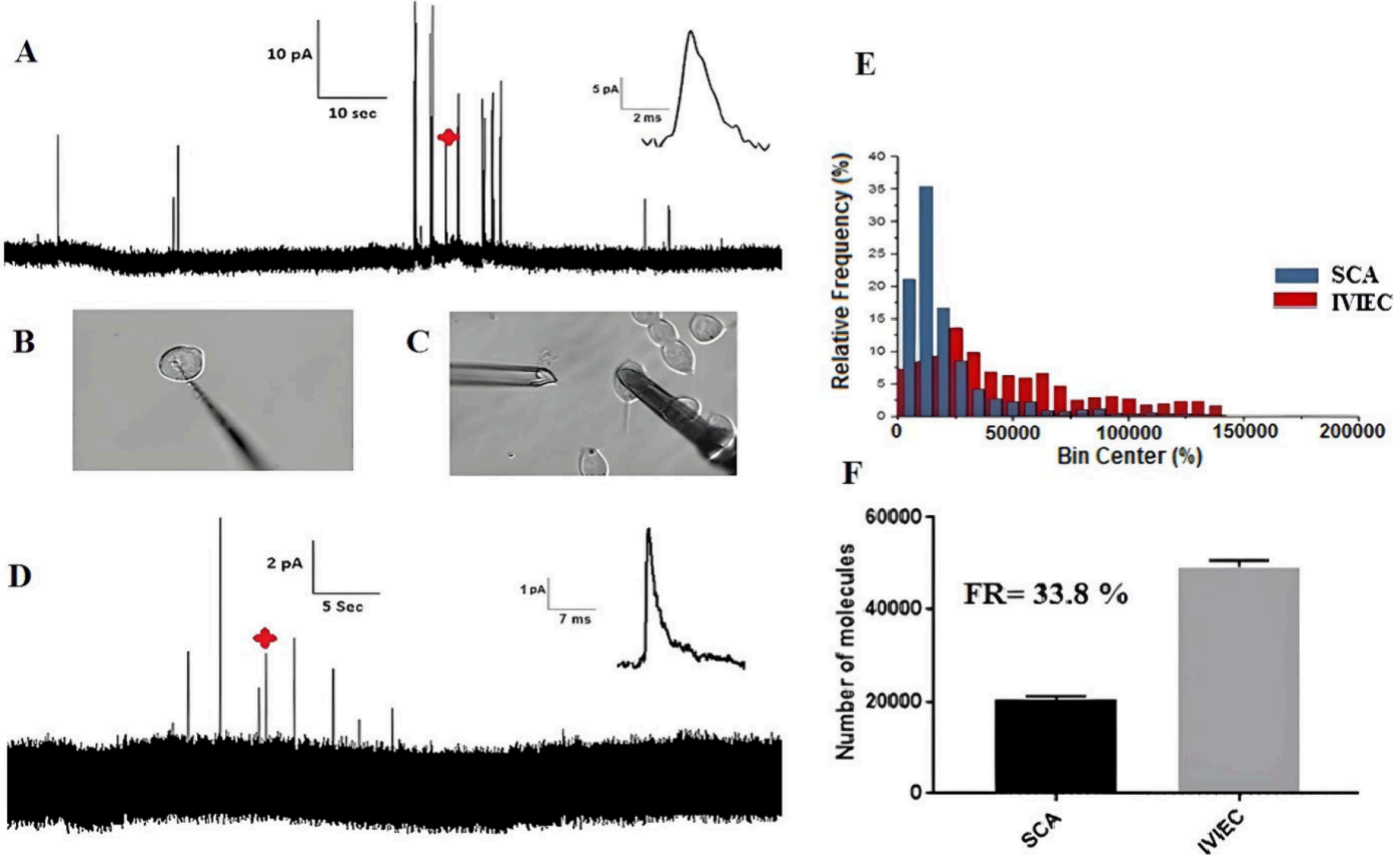
(**A**) Amperometric recording displaying the oxidation of preloaded 5-HT from vesicles of mouse primary beta cells using the intracellular vesicle impact electrochemical cytometry (IVIEC) technique (inset: amplified amperometric current spike indicated by a red star in the trace). (**B**) Diagrammatic representation showing the experimental configuration for the carbon nanotip electrode positioned inside a single cell for IVIEC measurements. (**C**) Diagrammatic representation of the carbon disk-shaped electrode on the surface of a single cell for SCA measurements. (**D**) Representative amperometric trace from SCA due to the oxidation of secreted 5-HT from a single primary mouse beta cell (inset: amplified amperometric current spike marked with a red star in the trace). (**E**) Histograms presenting the frequency of quantified secreted 5-HT from vesicles during exocytosis by SCA (Blue, Number of cells: 26) and the frequency of quantified stored 5-HT within vesicles by IVIEC techniques (Red, Number of cells: 51). (**F**) Plot showing the mean and median number of molecules detected by SCA and IVIEC (Inset: calculated Fraction of Release (FR) of 5-HT in the tested single mouse beta cell). (Reprinted from ref (^19^) copyright 2021 Wiley-VCH).

Partial release has now been confirmed in many cellular systems^19,57,70−73^ and was further tested in single pancreatic beta cells from INS-1 cells, preloaded with 5-HT, by Hatamie et al. in 2021.^19^ They conducted IVIEC analysis using a carbon nanotip electrode to estimate the number of stored 5-HT molecules in individual vesicles, which was approximately 39,317 ± 1,611 molecules after loading. Additionally, SCA measurements with a carbon disk electrode were used to determine the number of 5-HT molecules released during each individual exocytosis event, which was approximately 13,310 ± 1,127 molecules (Figure 4).

The comparison of release to content indicates that the vesicles of tested beta cells release only around 34% of their 5-HT content, retaining the rest for future exocytosis. These quantitative amperometric data clearly revealed that beta cells prefer partial release. Since 5-HT is coreleased with insulin, this behavior can therefore also be linked to insulin exocytosis and diabetes, making the finding valuable for diabetes and diabetic research. The observation that beta cell vesicles release only a portion of their content when insulin is needed to regulate glucose levels, especially in diabetic cases, opens an opportunity for new research into this disease. As 5-HT is smaller, it might be expected to have a higher likelihood of exiting the vesicle through the pore during exocytosis compared to the larger insulin molecules, which have a lower diffusion rate. Thus, partial release of 5-HT observed from beta cells might be even more significant for insulin content even when the body needs more insulin in the bloodstream. The implications for diabetic patients is highly important.

## Conclusion and Perspectives

Analytical methods provide novel information about insulin production, storage in vesicles, and the cellular mechanisms of β-cell stimulation and insulin secretion at subcellular levels. One such technique is single-cell amperometry with micronanoscale electrodes, which offers unique quantitative analysis with superior temporal and spatial resolution at the single-cell and even vesicle levels in real time. In the 1990s, this technique was successfully applied to count released insulin during exocytosis from single beta cells, enabling real-time measurement of released molecules and studying the effect of the chemical environment (such as the presence of extra levels of free Zn^2+^ and varying extracellular pH medium) on β-cell behavior and insulin secretion yield.^21,22^ This finding demonstrated the capability of single-cell amperometry in diabetes research, although the electroanalysis of insulin is challenging and requires modified electrodes. Recently, a new version of cell amperometry with nanoscale electrodes, called intracellular electrochemical cytometry, was developed. This allows accurate analysis of single vesicle content inside the cell before exocytosis. Combining single-cell amperometry and intracellular electrochemical cytometry provides a novel analytical strategy to examine the hypothesis often credited to Katz,^74,75^ which states that, excluding kiss n run release, regular exocytosis from intracellular vesicles is an all-or-none process, a mystery in the exocytosis process for decades. Results from applying a combination of these techniques to various cell types, particularly β-cells, indicate that partial release is the primary mode of exocytosis. These findings about β-cell behavior could increase our understanding of the dynamics of insulin storage and release from single vesicles, a phenomenon not reported with other methods. Further amperometric measurements of partial release in treated neural cells have shown that the fraction of partial release can be modulated. In the near future, this regulation of exocytosis in beta cells could be used to potentially increase the exocytosis rate for β-cells and benefit people with diabetes.

## Future Outlook

In recent decades, advances in nanotechnology and nanomicrofabrication have enabled the development of new nanoelectrodes which can enhance the capabilities of single-cell amperometry for other goals and applications.^76−78^ Future nanomicroelectrode systems may be capable of simultaneously measuring multiple parameters in a single β-cell, such as glucose, insulin, and other biomolecules and hormones like glucagon and somatostatin. Additionally, combining amperometric techniques with sensitive optical imaging, like STED microscopy, can provide imaging and electrochemical data simultaneously at the single-cell level, opening new views and possibilities in current investigations, particularly about vesicle structures and the dense core in beta cells. Furthermore, interdisciplinary collaboration among researchers in different fields could fill existing gaps, such as the combination of amperometry with artificial pancreas systems or the current lab-on-a-chip technologies^79,80^ to support and facilitate medical treatment research. In this area, we should not ignore artificial intelligence, which offers accurate and multiple data analyses and could revolutionize electroanalysis applications in diabetic investigations at the cellular level.

Importantly, in addition to providing the classic quantitative data discussed in this review, nanomicro amperometry tools and others will enable the acquisition of novel information, especially about the dynamics of exocytosis and the fusion pore, a tiny biogate where insulin leaves the vesicles during exocytosis. This technique can support careful testing on single β-cell function and unlock mechanistic information about diabetes drugs, for example, their effect on the fusion pore and varying insulin secretion yield.

## Abbreviations

SCA: single-cell amperometry
T2D: type 2 diabetes
CFME: carbon fiber microelectrodes
5-HT: or 5-hydroxytryptamine or serotonin
